# Targeting the Brain Leptin-Melanocortin Pathway to Treat Heart Failure

**DOI:** 10.1007/s11906-024-01318-z

**Published:** 2024-11-29

**Authors:** Ana C. M. Omoto, Jussara M. do Carmo, Alan J. Mouton, Zhen Wang, Xuan Li, Robert Spitz, John E. Hall, Alexandre A. da Silva

**Affiliations:** Department of Physiology and Biophysics, Cardiorenal and Metabolic Diseases Research Center, Mississippi Center for Obesity Research, University of Mississippi Medical Center, 2500 North State Street, Jackson, MS USA

**Keywords:** Central nervous system, Leptin receptors, Melanocortin 4 receptors (MC4R), Myocardial infarction

## Abstract

**Purpose of the Review:**

The role of leptin in regulating cardiac function is still controversial with conflicting results in clinical and preclinical studies. However, most previous studies have not considered leptin’s powerful cardiac effects that are mediated via activation of central nervous system (CNS) leptin receptors (LepRs) which, in turn, elicit major improvements in cardiac metabolism. In this review, we focus mainly on the role of leptin in regulating cardiac function via its CNS LepRs and downstream signaling pathways, such as the brain melanocortin system.

**Recent Findings:**

Studies from our laboratory showed that CNS LepR activation, without raising plasma leptin levels, has remarkable beneficial effects on cardiac metabolism and function that protect the heart during pathological conditions, including heart failure (HF) induced by myocardial infarction (MI). These cardioprotective effects of leptin appear to be mediated by stimulation of CNS proopiomelanocortin neurons and subsequent activation of melanocortin 4 receptors (MC4R) in the brain.

**Summary:**

Chronic activation of the brain leptin-melanocortin pathway improves cardiac function and metabolism following myocardial infarction. However, the mechanism underlying this brain-heart crosstalk remains unclear and may have important implications for the development of new therapies for MI and HF.

## Introduction

Leptin, a peptide hormone produced mainly by the white adipose tissue, informs the brain about the body’s energy status. Leptin is widely recognized for its capacity to reduce food intake and increase energy expenditure by acting as an afferent signal in a negative-feedback loop for body weight and energy stores homeostasis [1].

In healthy lean individuals, an increase in leptin levels almost invariably results in reduced food intake and increased energy expenditure, whereas decreases in leptin levels have opposite effects. This circuit of afferent and efferent signals between brain and adipose tissue maintains the stability of energy stores, which is essential for survival, especially in extreme situations such as starvation [1]. Although individuals with obesity have elevated levels of leptin, they may be at least partially resistant to leptin’s metabolic effects [2, 3]. Despite having high levels of leptin, these individuals continue to overeat and leptin’s effect to increase energy expenditure is blunted [4].

Given the strong association between obesity and cardiovascular diseases, and the fact that obese individuals are hyperleptinemic, in this mini review we summarize some of the conflicting studies related to leptin's impact on cardiac function and recent studies that have discovered novel cardioprotective effects of activating the brain leptin-melanocortin pathway following ischemic injury.

## Conflicting Observations on the Impact of Leptin on Cardiac Function

The impact of leptin on cardiac function has been extensively investigated with conflicting results [5–9]. For example, epidemiological studies showed that increased plasma leptin levels correlate positively with adverse cardiovascular outcomes, such as myocardial infarction (MI) and congestive heart failure (HF) [10–12]. However, these studies were not designed to examine cause-and-effect relationships. Additionally, since individuals with elevated plasma leptin are often obese, it is challenging to isolate the effects of hyperleptinemia from other obesity-related adverse influences on cardiac structure and function. Thus, it remains uncertain whether high plasma leptin levels are directly linked to higher risk of developing HF.

Alternatively, preclinical studies using leptin-deficient animals (*ob/ob* mice) demonstrated that restoring plasma leptin concentration to normal improved heart function after MI, and that these beneficial effects were associated with reductions in cardiac inflammation and apoptosis [13, 14] and activation of anti-hypertrophic pathways in obese animals with left ventricle (LV) hypertrophy [8]. Induction of cardiac specific leptin receptor (LepR) deficiency in lean adult mice also caused severe cardiac dysfunction [15, 16]. Furthermore, rescue of LepRs in cardiomyocytes of obese *db/db* mice (animals with global LepR deficiency) improved heart function and attenuated cardiac lipotoxicity [6].

Other preclinical studies, however, showed that exposure of cultured neonatal cardiomyocytes to high levels of leptin increased cardiomyocyte cell surface area, suggesting a hypertrophic effect [17, 18], and administration of a LepR antagonist in the ascending aorta attenuated LV hypertrophy induced by ascending thoracic aorta aneurysm in angiotensin II-infused mice [19]. Purdham et al. showed that chronic administration of a LepR-neutralizing antibody improved cardiac function and remodeling in a rat model of MI [20]. Thus, conflicting observations have been reported for the direct impact of leptin on the heart.

## CNS Leptin Activation and Its Effects in Cardiac Function and Metabolism

Although the impact of leptin acting via direct effects on cardiac LepRs is still controversial, most, if not all, studies examining the effects of leptin on cardiac metabolism, structure, and function evoked by activation of CNS LepRs indicate beneficial effects. [9, 21]. For instance, administration of leptin into the brain ventromedial hypothalamus stimulated glucose uptake and oxidation in multiple tissues, including the heart, and this effect appeared to be independent of insulin since CNS leptin administration did not increase plasma insulin levels [22]. Studies from our laboratory also showed that chronic intracerebroventricular (ICV) infusion of leptin improved intrinsic heart rate and reversed the bradycardia associated with diabetic cardiomyopathy in insulin-deficient rats [23].

Corroborating the concept that leptin, via its actions on the CNS, exerts an important beneficial effects on cardiac metabolism, Sloan et al. demonstrated that CNS leptin administration normalized myocardial fatty acid oxidation, and improved glucose oxidation and mitochondrial function in hearts of leptin-deficient *ob/ob* mice, and this effect was independent of caloric intake and reduction in body weight [24]. Also, Mora et al. demonstrated that CNS leptin administration increased lipolysis and reduced lipogenesis, leading to improved control of triacylglycerol (TAG) content and reduced lipotoxicity in the heart [25].

**Fig. 1:**
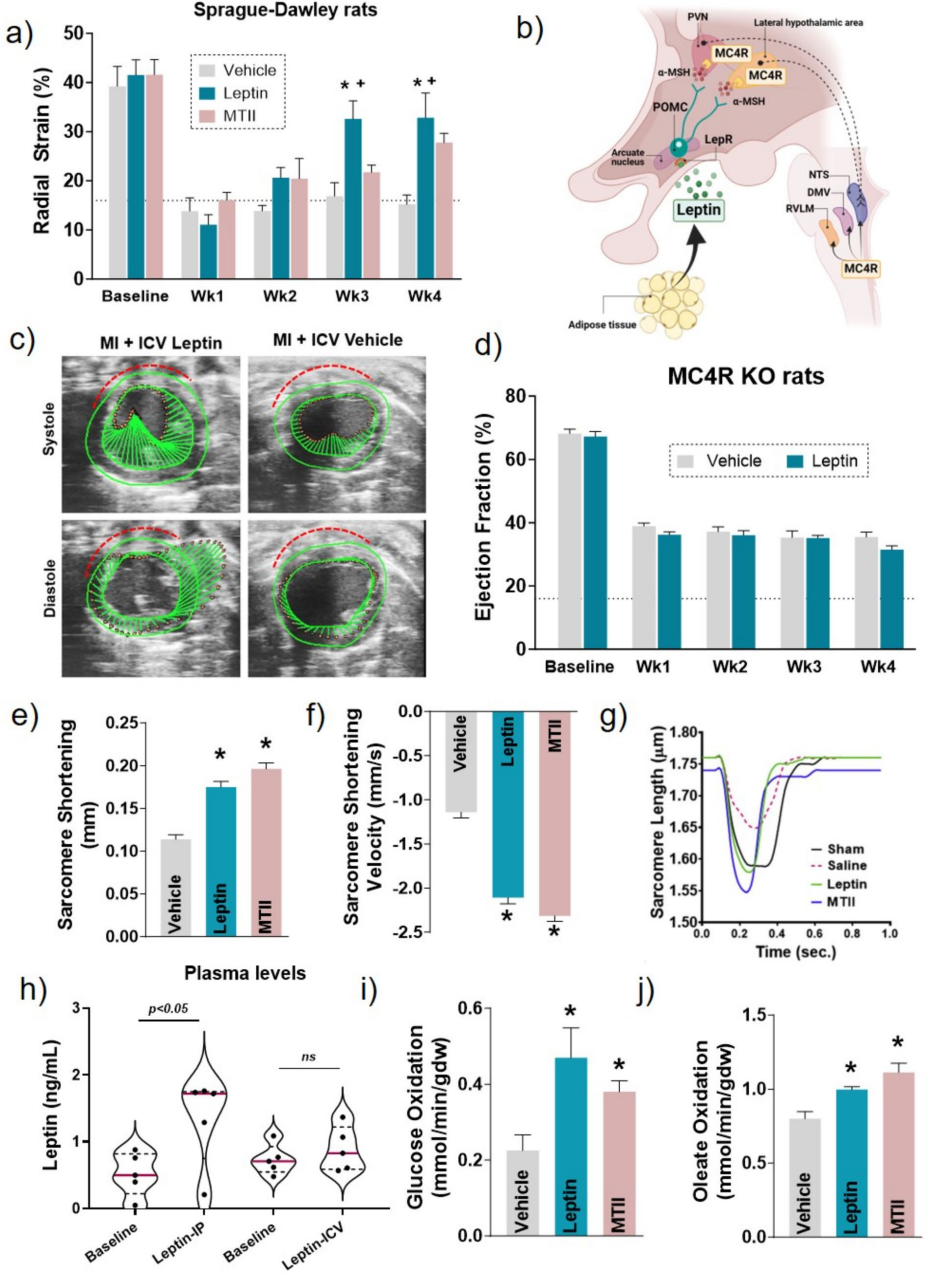
Activation of the brain leptin-melanocortin pathway have cardioprotective effects in MI-induced HF. **a**) Radial strain in Spraque-Dawley rats treated with ICV leptin or MTII; **b**) schematic drawing of the brain-melanocortin pathway; **c**) representative figure of radial strain in a short axis view of the left ventricle where the size of the green vectors represents cardiac fibers deformation during systole and diastole and the red dotted line delineate the infarct size; **d**) ejection fraction in MC4R knock out rats treated with ICV leptin or vehicle; **e** – **g**) isolated cardiomyocytes contractility; **h**) plasma leptin levels in animals that received IP or ICV leptin; **i** – **j**) measurements of glucose and fatty acid oxidation using working heart preparation. ICV: intracerebroventricular, IP: intraperitoneal, MTII: melanotan II, MC4R: melanocortin 4 receptor, PVN: paraventricular nucleus, α-MSH: α-melanocyte hormone, POMC: proopielomelanocortin, LepR: leptin receptors. * vs vehicle *p* < 0.05; + vs MTII, *p* < 0.05

**Fig. 2:**
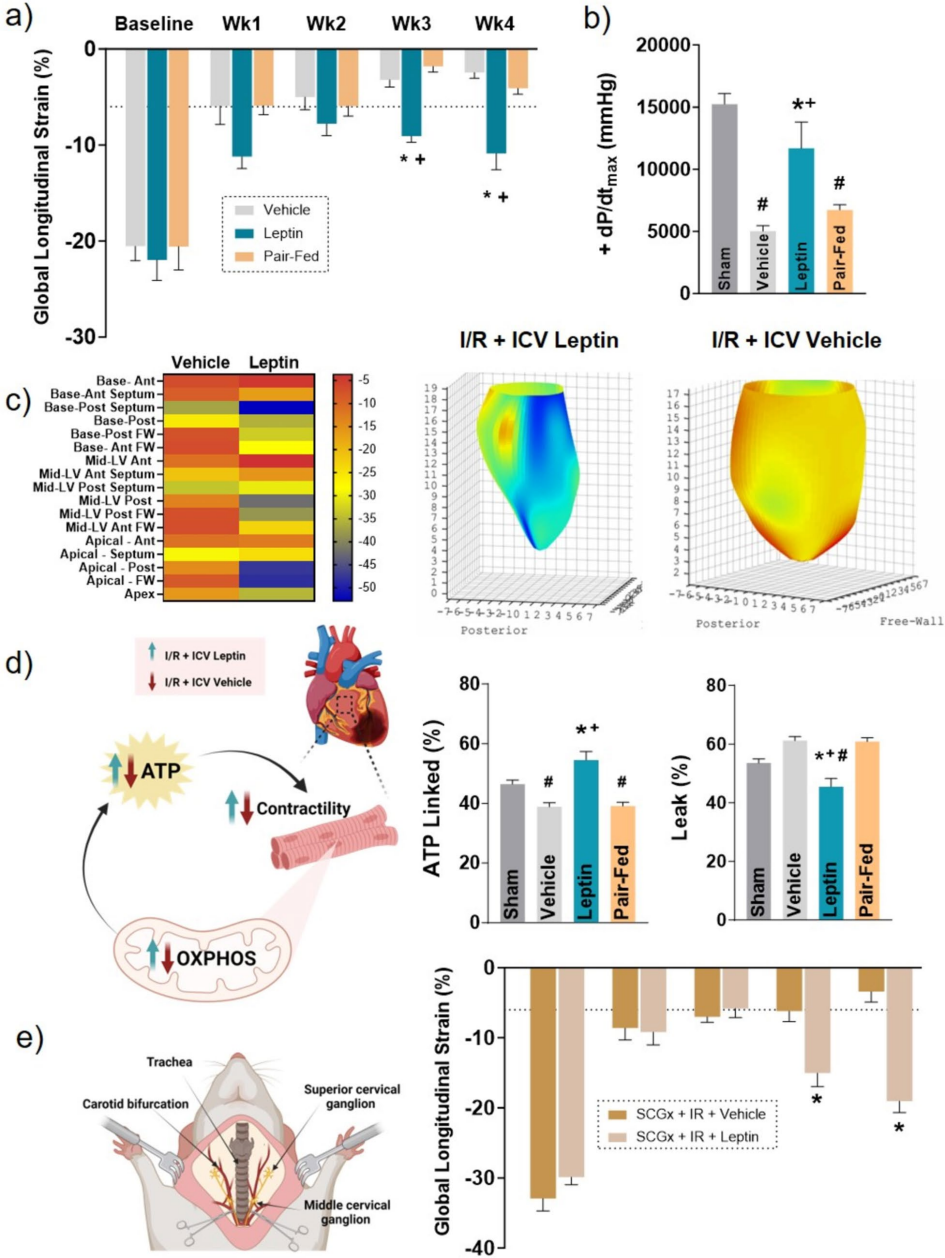
CNS leptin administration improves cardiac and mitochondrial function regardless of reductions in food intake and cardiac sympathetic denervation. **a**) global longitudinal strain; **b**) left ventricular pressure; **c**) heat map representation of regional endocardial surface strain and a 4-dimensional reconstruction of the endocardial surface strain in vehicle and leptin-treated rats after myocardial I/R; **d**) mitochondria function measured in isolated cardiac fibers using Oroboros _2_Oxygraphy-2k; **e**) schematic representation of sympathetic cervical ganglia denervation (SCGx) and global longitudinal strain measured in animals with SCGx treated with vehicle or leptin post myocardial I/R. I/R: ischemia–reperfusion injury; dP/dt_max_: maximal rate of left ventricle pressure rises; SCGx: sympathetic cervical ganglia denervation; ICV: intracerebroventricular, ATP: adenosine triphosphate; OXPHOS: oxidative phosphorylation. * vs vehicle *p* < 0.05; + vs pair-fed, *p* < 0.05; # vs sham, *p* < 0.05

Recently, our laboratory demonstrated that chronic CNS leptin infusion improved cardiac metabolism and function and attenuated progression of HF after MI induced by permanent ligation of the left descending coronary artery (Fig. 1) or ischemia followed by reperfusion (I/R) (Fig. 2) [26, 27]. We showed that leptin, via its CNS actions, improved radial strain (Figs. 1a and c), cardiomyocyte contractility (Figs. 1e**–**g), and stimulated glucose and fatty acid oxidation in non-infarcted regions of the heart (e.g. septum) (Figs. 1i and j) [27]. In a subsequent study, our laboratory showed that CNS leptin activation also improved global longitudinal strain and surface area strain (Figs. 2a and c), cardiac contractility measured by left ventricle catheterization (Fig. 2b), and mitochondrial function in isolated cardiac fibers (Figs. 2d and e) in a model of I/R injury [26]. Importantly, these findings were independent of any direct action of leptin in the heart since no spillover of leptin into the circulation was detected during ICV leptin infusion (Fig. 1h). Collectively, these studies indicate that activation of leptin pathways in the CNS elicits remarkable cardioprotective effects after ischemic injury.

## Brain Leptin-Melanocortin System

One key action of leptin in the CNS is to stimulate the brain melanocortin system which is involved in the control of energy balance, food intake and body weight homeostasis [2]. Leptin binds to its receptors in proopiomelanocortin (POMC)-containing neurons located mainly in the arcuate nucleus of the hypothalamus and in the brainstem. Leptin stimulates the release of α-melanocyte–stimulating hormone (α-MSH) by neuronal projections of POMC neurons in various regions of the CNS including the paraventricular nucleus and lateral nucleus of the hypothalamus (Fig. 1b). The released α-MSH activates melanocortin 3 and 4 receptors (MC3R and MC4R) in the hypothalamus and brainstem [28, 29].

Along with regulation of energy balance, the brain leptin-melanocortin system axis also regulates cardiovascular function, including blood pressure (BP) and heart rate (HR), by stimulating sympathetic activity and inhibiting parasympathetic nervous system activity [2, 28, 30]. POMC neurons are also present in the nucleus of the tractus solitarius (NTS) in the brain stem, an important brain region involved in autonomic control [29].

In addition to its effects on sympathetic activity, BP and HR, our laboratory showed that the brain melanocortin system, particularly MC4R, plays a critical role in contributing to leptin’s cardioprotective effects (Fig. 1). We demonstrated that long-term activation of MC4R using melanotan II (MTII), an MC4R agonist, recapitulates the cardioprotective effect of leptin after MI (Figs. 1a and 1c), and that genetic disruption of MC4R markedly attenuated or abolished leptin’s ability to improve cardiac metabolism and function after MI-induced HF (Fig. 1d) [27]. These findings highlight an important translational potential for MC4R agonists as a new therapeutic approach to treat MI and slow/halt HF progression after cardiac ischemia. MC4R agonists that can cross the blood–brain barrier, and thus can be administered systemically, are already approved to treat rare forms of genetic obesity in humans, and obese individuals are more susceptible to develop MI and HF.

## Brain–Heart Crosstalk During Activation of CNS Leptin-melanocortin Pathway: Possible Mediators

### Sympathetic Nervous System

Activation of the brain leptin-melanocortin system can increase sympathetic outflow to peripheral tissues and organs [2, 28]. In a recent study, we tested the hypothesis that leptin-induced sympathetic activation contributes to the improvement of cardiac contractility and function in rats with I/R-induced HF by performing cervical gangliectomy to reduce cardiac sympathetic innervation during ICV leptin treatment (Fig. 2). We found that cardiac function was still improved by CNS leptin treatment in rats with I/R-induced HF (Fig. 2e) [26], suggesting that cardiac sympathetic fibers may not be a major mediator of the cardioprotective effects elicited by CNS leptin infusion.

Although the cervical sympathetic ganglia heavily innervates the heart and we observed a marked reduction in sympathetic innervation to cardiac tissues [26], other sources of sympathetic innervation to the heart were preserved (e.g., the stellate ganglia) which could still be involved in contributing to the crosstalk between the brain and heart in response to CNS leptin treatment. Additional studies with complete sympathetic denervation to the heart (including the stellate ganglia) or using transgenic animal models with specific deletion of adrenergic receptors in the heart are needed to fully examine the role of cardiac sympathetic nerves.

It is also important to note that although cardiac sympathetic fibers denervation did not markedly attenuate the beneficial effect of CNS leptin administration on cardiac metabolism and function post-I/R, leptin-induced increase in sympathetic activity to other peripheral organs may indirectly mediate the cardioprotective effects elicited by CNS leptin infusion.

### Brown Adipose Tissue

Brown adipose tissue (BAT) is also richly innervated by sympathetic fibers and its sympathetic activity is increased by CNS leptin administration. Previous studies demonstrated that administration of leptin in hypothalamic nuclei increases glucose uptake by BAT, raises BAT temperature, and increases UCP1 expression [31–33], the main protein involved in mitochondrial uncoupling and heat generation in BAT. These findings indicate that CNS leptin administration increases BAT activity.

Recent studies have identified an important endocrine/paracrine role for the BAT in regulating the function of a variety of organs, including the heart, through BAT-derived molecules, also termed “batokines” [34]. In addition, there is evidence that extracellular vesicles (EVs) released from BAT contribute to exercise-induced cardioprotection after I/R injury, and that BAT-derived EVs carry microRNAs that suppress pro-apoptotic pathways and protect the heart against I/R injury [35].

Previous studies also revealed the importance of POMC neurons and MC4R in regulating BAT thermogenesis [36, 37]. For example, Satoh et al. showed that CNS administration of the MC4R antagonist, SHU9119, completely blocked leptin-induced increase in BAT UCP1 mRNA expression [38]. Williams et al. demonstrated that CNS administration of the MC4R agonist, MTII, increased BAT UCP1 mRNA expression, and that this increase could be blocked by BAT sympathetic denervation [39]. In addition, transgenic mice with whole-body MC4R deficiency exhibit impaired upregulation of BAT UCP1 in response to cold exposure when compared to wild-type controls [40]. Together these studies highlight the importance of the leptin-melanocortin system axis in controlling BAT activity and suggest that the brain melanocortin system is a key downstream pathway by which leptin modulates sympathetic activity to BAT and BAT function, and possibly BAT production and secretion of batokines.

Preliminary data from our laboratory [41] indicate that surgical removal of BAT or BAT sympathetic denervation attenuates the improvement of cardiac function evoked by CNS leptin infusion in rats with MI-induced HF. These studies suggest a possible interorgan crosstalk including brain-BAT-heart that is modulated by LepR activation in the CNS, and this may constitute a novel target for therapies to protect the heart against ischemic insults.

### Cholinergic Anti-inflammatory Pathway

The brain exerts a strong modulatory effect on the immune system. For example, activation of the hypothalamus–pituitary–adrenal (HPA) axis by cytokines (e.g., IL-1 and IL-6) triggers synthesis and release of glucocorticoids with potent immunosuppressor effects [42].

The vagus nerve has also been proposed to participate in an immunomodulatory circuit with systemic and local anti-inflammatory properties. Borovikova et al. demonstrated that electrical stimulation of the vagus nerve during severe endotoxemia inhibited TNFα synthesis and attenuated development of endotoxin shock in rats [43]. They also showed that macrophages express the α7 subunit of the nicotinic acetylcholine receptor which mediates the anti-inflammatory activity of the vagus nerve [43].

Previous studies suggest that leptin may also regulate the immune response via its CNS actions. Tschop et al. showed, for example, that CNS leptin administration increases survival in an animal model of sepsis, and this effect was not a consequence of leptin-induced modulation of the HPA axis since plasma levels of corticosterone were not altered in leptin treated animals [44]. The authors also showed that rescuing LepR specifically in the CNS of *db/db* mice improved survival during sepsis [44]. Since corticosterone levels were not elevated in these animals, they speculated that CNS leptin administration likely attenuates inflammation through activation of the anti-inflammatory cholinergic pathway.

Although leptin is known to increase sympathetic activity, LepRs are also expressed in cholinergic neurons of the dorsal motor nucleus of the vagus [45], a structure containing important preganglionic motor neurons that modulate several visceral functions.

Preclinical studies also indicate that the brain melanocortin system can control peripheral inflammation. For example, administration of α-MSH directly into the brain inhibits the acute inflammation caused by picryl chloride [46], blocks the immunosuppressive effect of central IL-1 administration [47], and reduces systemic inflammation in endotoxemic mice [48]. In addition, Bazzani et al. showed that central administration of adrenocorticotropin (ACTH-(1–24)), a melanocortin peptide, prevented the occurrence of ventricular tachycardia and fibrillation after I/R injury [49]. Collectively, these studies suggest another possible mechanism by which chronic CNS leptin-melanocortin axis activation improves cardiac function after MI. However, further studies are needed to examine this possibility.

### Neuroendocrine Factors

It is also possible that a circulating factor (or factors) released by the brain in response to activation of the CNS leptin-melanocortin pathway may contribute to the cardioprotective actions of leptin. Parabiosis studies from our group showed that central leptin infusion restores normoglycemia in type-1 diabetic rats and reduces blood glucose levels by ~ 27% in the conjoint parabiotic rat receiving only vehicle in the brain [50]. This finding indicates that leptin acts on the CNS to stimulate the release of a circulating transferable factor that enhances glycemic control in diabetic conjoined parabiotic rats and could also be involved in the cardioprotective effect of central leptin infusion after MI.

This potential neurohumoral factor with strong antidiabetic effects does not appear to require normal pituitary function since hypophysectomy did not abolish the normalization in glucose levels during CNS leptin infusion in type-1 diabetic animals [51]. However, hypophysectomy attenuated leptin’s effect to raise heart rate [51]. Whether pituitary hormones are also important for the cardioprotective effects of central leptin-melanocortin activation remains uncertain and awaits further investigation.

Other neuronal factors could also be involved in the cardiac protection elicit by CNS leptin. For example, the brain derived neurotrophic factor (BDNF), a neurotrophin highly expressed in the hypothalamus and other brain centers involved in homeostatic regulation of energy balance, seems to ameliorate the diabetic and obese phenotype of LepR deficient mice [52–54]. Also, Okada et al. demonstrated that brain-specific BDNF conditional knockout mice present worse cardiac remodeling and function after MI compared with control mice suggesting a cardioprotective role of neuronal BDNF production after MI [55]. However, further investigation is needed to evaluate whether neuronal BDNF signaling is necessary for the cardioprotective effect elicited by CNS leptin administration after MI.

### Perspectives

Ischemic heart diseases account for ~ 50% of all cardiovascular deaths and the search for therapies that improve cardiac function after MI has been the focus of many researchers over the years. Recent studies demonstrating interorgan crosstalk during pathologic conditions, including cardiac ischemia, have provided promising new therapeutic strategies for MI as well as for protecting other organs after ischemic stress.

**Fig. 3:**
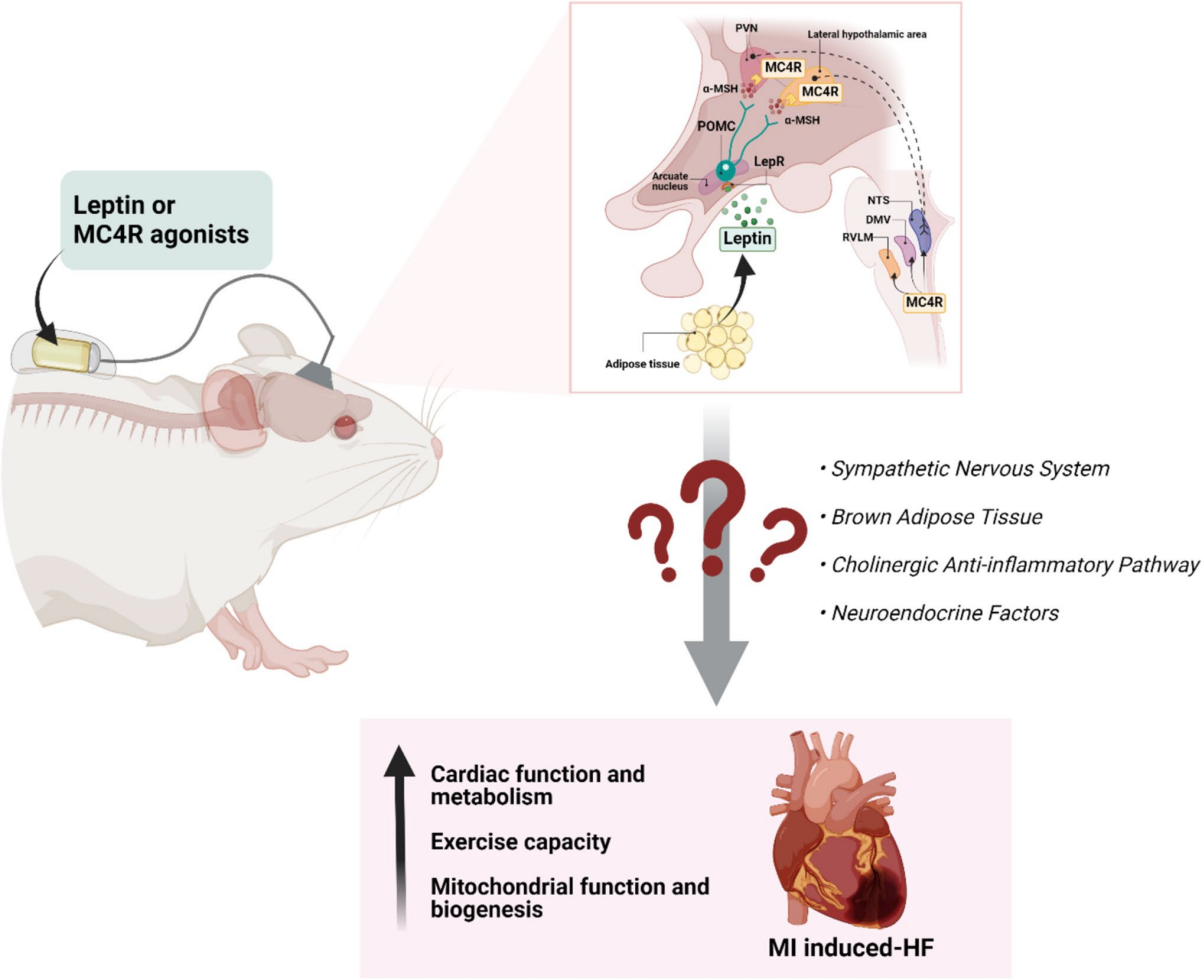
Cardioprotective effects of the brain leptin-melanocortin pathway activation. Chronic intracerebroventricular infusion of leptin or melanocortin 4 receptor (MC4R) agonists improve cardiac function and metabolism in a rat model of heart failure induced by myocardial infarction (MI). Leptin and MC4R agonists also improve mitochondrial function and biogenesis in non-infarcted areas of the heart (e.g. septum) and enhance exercise capacity. However, the mediators involved in the brain–heart crosstalk during CNS leptin-melanocortin activation are still unknown. Some potential candidates include the sympathetic nervous system, factors released from brown adipose tissue, the cholinergic anti-inflammatory pathway, and neuroendocrine factors

Stimulation of the CNS leptin-melanocortin system by directly infusing leptin or MC4R agonists into the brain protects the heart after MI (Fig. 3). Moreover, the cardioprotective effects of leptin appear to be mediated almost entirely by activation of POMC neurons and ultimately activation of MC4R. Although direct CNS administration of leptin or MC4R agonists may not be feasible for clinical studies or therapy for MI in humans, new MC4R agonists, such as setmelanotide, cross the blood brain barrier and can be administered peripherally and are already approved by the FDA to treat rare forms of genetic obesity. How this cardiac protective effect of stimulating the brain leptin-melanocortin pathway is transmitted to the heart (Fig. 3) and its long-term effects (beyond 4 weeks) on cardiac function after MI are still being explored, and further studies are needed to assess their therapeutic potential in humans after MI or ischemic insults in other organs. This emerging area of interorgan crosstalk research shows promise and warrants greater attention.

## Key References

- Omoto ACM, do Carmo JM, Nelson B, Aitken N, Dai X, Moak S et al. Central Nervous System Actions of Leptin Improve Cardiac Function After Ischemia–Reperfusion: Roles of Sympathetic Innervation and Sex Differences. J Am Heart Assoc. 2022;11(21):e027081. 10.1161/jaha.122.027081.
  - ⚬ Findings from this study showed that chronic intracerebroventricular (ICV) of leptin, with no spill over in the circulation, improved cardiac function after ischemia/reperfusion injury in male and female rats and that this effect is not transmitted from the brain to the heart by cardiac sympathetic nerves.
- Gava FN, da Silva AA, Dai X, Harmancey R, Ashraf S, Omoto ACM et al. Restoration of Cardiac Function After Myocardial Infarction by Long-Term Activation of the CNS Leptin-Melanocortin System. JACC Basic Transl Sci. 2021;6(1):55–70. 10.1016/j.jacbts.2020.11.007.
  - ⚬ Findings from this study showed that ICV infusion of leptin improved cardiac function after myocardial infarction (MI) in rats, an effect that seems to be mediated by the brain melanocortin 4 receprtos (MC4R) since the cardioprotective effect of ICV leptin infusion is lost in MC4R knockout rats and infusion of MC4R agonist in the brain recaptulates leptin’s cardioprotective effect post-MI.
- da Silva AA, Hall JE, Dai X, Wang Z, Salgado MC, do Carmo JM. Chronic Antidiabetic Actions of Leptin: Evidence From Parabiosis Studies for a CNS-Derived Circulating Antidiabetic Factor. Diabetes. 2021;70(10):2264–74. 10.2337/db21-0126.
  - ⚬ Findings from this study showed that chronic ICV leptin infusion, at rates that did not increase plasma leptin levels, restored normoglycemia in streptozotocin (STZ)–induced diabetic rats and reduced blood glucose concentration in the conjoined parabiotic rats infused with vehicle.

## Data Availability

No datasets were generated or analysed during the current study.
